# Diagnostic Accuracy of Pulp Vitality Tests and Pulp Sensibility Tests for Assessing Pulpal Health in Permanent Teeth: A Systematic Review and Meta-Analysis

**DOI:** 10.3390/ijerph19159599

**Published:** 2022-08-04

**Authors:** Swadheena Patro, Agron Meto, Ankita Mohanty, Viresh Chopra, Sanjay Miglani, Antarikshya Das, Alexander Maniangat Luke, Dunia Al Hadi, Aida Meto, Luca Fiorillo, Mohmed Isaqali Karobari, Dian Agustin Wahjuningrum, Ajinkya M. Pawar

**Affiliations:** 1 Department of Conservative Dentistrty and Endodontics, Kalinga Institute of Dental Sciences, KIIT University, Bhubaneswar 751024, India; 2 Department of Dentistry, Faculty of Dental Sciences, University of Aldent, 1007 Tirana, Albania; 3 Department of Adult Restorative Dentistry, Oman Dental College, Muscat 116, Oman; 4 Department of Conservative Dentistrty and Endodontics, Faculty of Dentistry, Jamia Millia Islamia (A Central University), Okhla, New Delhi 110025, India; 5 Department of Clinical Sciences, College of Dentistry, Ajman University, Ajman P.O. Box 346, United Arab Emirates; 6 Centre of Medical and Bio-Allied Health Sciences Research, Ajman University, Ajman P.O. Box 346, United Arab Emirates; 7 Department of Dentistry, Faculty of Dental Medicine, University of Western Balkans, 1051 Tirana, Albania; 8 Department of Biomedical and Dental Sciences, Morphological and Functional Images, University of Messina, 98100 Messina, Italy; 9 Multidisciplinary Department of Medical-Surgical and Odontostomatological Specialties, University of Campania “Luigi Vanvitelli”, 80121 Naples, Italy; 10 Department of Restorative Dentistry & Endodontics, Faculty of Dentistry, University of Puthisastra, Phnom Penh 12211, Cambodia; 11 Department of Conservative Dentistry, Faculty of Dental Medicine, Universitas Airlingga, Surabaya City 60132, Indonesia; 12 Department of Conservative Dentistry and Endodontics, Nair Hospital Dental College, Mumbai 400008, India

**Keywords:** dental pulp, dental pulp test, dentistry, pulp vitality, pulse oximeter

## Abstract

The current systematic review and meta-analysis was carried out to compare the diagnostic accuracy of pulp vitality and pulp sensibility tests in assessing pulpal health. PubMed/MEDLINE, Cochrane Central Register of Controlled Trials, Web of Science, Google Scholar and Open Grey databases were searched and after assessing eligibility criteria the data were extracted. True-positive, false-positive, true-negative, false-negative, sensitivity and specificity values were extracted or calculated if not presented. Quality of studies was evaluated based on the QUADAS 2 tool. Meta-analysis was performed in MetaDTA (v2.0; Shinyapps, RStudio PBC, Boston, MA, USA) and Review Manager 5.3 (RevMan web; The Cochrane Collaboration, London, UK). Ten articles were included for qualitative synthesis and five for meta-analysis. The pooled diagnostic odds ratio for pulse oximeter (PO), electric pulp tester (EPT), cold test (CT) and heat test (HT) was 628.5, 10.75, 17.24 and 3.47, respectively. Pairwise comparison demonstrated a higher pooled mean sensitivity and specificity with PO compared with EPT. Comparison between PO and CT and between PO and HT also demonstrated a higher pooled mean sensitivity and specificity for PO. Summary points on receiver operating characteristic curves confirmed the ability of PO to correctly screen negatives in presenting patients as compared to EPT, CT and HT but no study was rated as good on quality assessment. PO can be considered as the most accurate diagnostic method as compared to EPT, CT and HT. This review provides information about the reliability and diagnostic accuracy of using pulp vitality and sensibility tests for assessing pulp status.

## 1. Introduction

In endodontics, dental pulp testing is a significant and essential diagnostic aid since diagnosis is an integral aspect of treatment planning. [1]. The gold standard of determining vitality status of pulp is directly inspecting it by histological section examination. However, as the pulp is enclosed by a calcified barrier, this cannot be carried out before starting endodontic therapy [2].

Inflammatory mediator components found in pulps exposed to caries or other lesions, such as prostaglandins, superoxide dismutase, tumor necrosis factor alpha (TNF-α), substance P and matrix metalloproteinases (MMPs), may indicate pulp state and can predict the outcome of pulp capping or pulpotomy treatments. When the pain presentation is inconsistent and abnormal, with the potential of referred or nonodontogenic pain, pulp testing can aid in accurate diagnosis through a confirmation or exclusion procedure. Changes in intra-pulp pressure have a significant impact on sensory nerves of varying dimensions, with pressure increases preferentially blocking larger diameter A-delta fibers and activating smaller diameter C-fibers. As C-fibers are more resistant to hypoxia, they may still function when the pulp degenerates due to the underlying pathology. When there is a complete absence of response to a stimulation, pulp necrosis is likely to have advanced. It should also be highlighted that the subjective nature of pain, individual variability in pain threshold and pain modulation processes make it difficult to obtain an exact history of clinical symptoms [1,3].

Since the pulp tissue cannot be directly inspected, indirect methods that determine the state of pulpal health by assessing the condition of the nerves within the dental pulp, such as pulp sensibility tests, must be employed. The most commonly used pulp sensibility tests are thermal tests and electrical tests that stimulate the pulpal nerves either by the flow of dentinal fluid at temperature variations, which leads to movement of the odontoblast processes and consequently mechanically stimulating the pulpal nerves, or by conducting electrical current through the tooth, giving an electrical stimulation to the nerves of the pulp [4,5].

The principal mechanism of the electric pulp test is to instigate an ionic change across the neural membrane by electrical stimuli which influences action potential with a fast-jumping action at the nodes of Ranvier in myelinated nerves [6]. The current pulp sensibility testing methods indirectly monitor pulp vitality by merely assessing the neural response and do not take into account the vascular circulation, resulting in false-positive responses for teeth that have temporarily or permanently lost their sensory function and are nonresponsive to these tests despite having an intact vasculature [7,8,9]. The limitations of pulp sensibility testing were overcome by pulp vitality testing methods such as pulse oximetry (PO), laser Doppler flowmetry (LDF) and ultrasound Doppler flowmetry (UDF) which assess pulpal blood flow without relying on the patients’ responses and are thought to deliver more accurate pulp status [10,11,12]. PO assesses the oxygen saturation inside the pulp chamber using a noninvasive catheter with two diodes adjusted to the teeth whereas LDF and UDF assess the vascular flow of the dental pulp through “the concentration and velocity of blood cells”, reflecting the signs of blood flow and pulp vitality [13,14,15].

Due to the obvious technological difficulties, there have been conflicting interpretations of the accuracy of pulp testing using PO and LDF. PO requires custom-made probes, and interferences due to the overhead xenon arc lamps as well as excessive carbon dioxide in the bloodstream may interfere with deoxygenation values, resulting in false results. In the case of LDF, when the laser pathway is interfered with or obstructed, false results may be achieved suggesting no blood flowing in that region. Similarly, the amount of signal contamination or noise from nonpulp sources, primarily the periodontium, may suggest the presence of pulp blood flow, leading to false readings [16,17]. Considering that there is currently no evidence supporting the use of pulp vitality testing over sensibility tests, a qualitative and quantitative synthesis of previously performed diagnostic accuracy studies is warranted.

Sensitivity and specificity best define the validity of a diagnostic test, while its clinical usefulness in a given population is best described by its positive and negative predictive values (PPVs and NPVs) [18]. Sensitivity is the proportion of cases identified correctly using the diagnostic test whereas specificity is the proportion of noncases identified correctly using the diagnostic test. Meanwhile, the positive predictive value is the proportion of positive test results that are cases and negative predictive value is the proportion of negative test results that are noncases [19].

A systematic review and meta-analysis diagnostic that focused on the accuracy of cold pulp testing (CPT), heat pulp testing (HPT), electric pulp testing (EPT), LDF and PO has been published by Mainkar and Kim, and concluded that LDF and PO were the most accurate diagnostic methods and HPT was the least accurate diagnostic method [19]. The review was based on searches conducted till 2016 but no comparative evaluation between pulp vitality and pulp sensibility test was conducted. Lima et al. [15] also conducted a systematic review to evaluate the efficacy of vitality tests (PO and LDF) in the pulpal diagnosis of traumatized teeth in comparison with sensibility tests. In this review, only traumatized teeth were included and it was based on searches conducted till 2018, but no quantitative analysis was conducted. A preliminary electronic search revealed that since their publication, many more studies comparing the diagnostic accuracy of pulp vitality and pulp sensibility tests have been published.

Therefore, this study aimed to perform a systematic review and meta-analysis of clinical studies to assess and compare the diagnostic accuracy of pulp vitality and pulp sensibility tests in assessing pulpal health in permanent teeth.

## 2. Materials and Methods

### 2.1. Protocol and Registration

This systematic review and meta-analysis were registered in PROSPERO (CRD42020213741) and conducted according to the Preferred Reporting Items for Systematic Review and Meta-Analyses (PRISMA) statement [20]. The following focused question in the patient, intervention, comparison and outcome (PICO) format was proposed: “Is there a difference in the diagnostic accuracy of pulp vitality and pulp sensibility tests in assessing pulpal health in permanent teeth”?

### 2.2. Search Strategy

A comprehensive electronic search was carried out on databases, such as PubMed/MEDLINE, Cochrane Central Register of Controlled Trials and Web of Science until December 2020 to retrieve articles in the English language. A specific electronic search of journals, presented in Table 1, was conducted. The searches in the clinical trials database, cross-referencing and searches of gray literature were conducted using Google Scholar, Greylist and OpenGrey. Medical subject headings (MeSH) terms, keywords and other free terms combined with Boolean operators (OR, AND) were used for searching articles. The identical keywords were used for all search platforms following the syntax rules of each database. The search strategy and population, interventions, comparisons, outcomes and study design (PICOS) tool are presented in Table 1.

### 2.3. Inclusion Criteria Outline According to the PICOS Strategy

Population (P): Studies with patients having at least one permanent tooth in the mandibular or maxillary region, having carious teeth, symptomatic or asymptomatic irreversible pulpitis (IP) that needed endodontic access, traumatized teeth irrespective of sex, age, race or socioeconomic status. As reference standards, histologic analysis, direct clinical observation (access cavity) or presence of root canal filling (only to confirm nonvital teeth) to confirm the pulp diagnosis of a study sample were used.

Interventions (I): Studies assessing the diagnostic accuracy of pulp vitality tests (laser Doppler flowmetry (LDF), ultrasound Doppler flowmetry, pulse oximetry, thermometry) in permanent teeth.

Comparison (C): Studies assessing the diagnostic accuracy of pulp vitality tests (thermal (hot or cold), electric pulp tester) in permanent teeth.

Outcome (O): The main outcome measures of this systematic review were to assess the pool estimates of sensitivity, specificity, PPV, NPV, likelihood ratio (LR) and diagnostic odds ratio of individual test groups as well as to compare the vitality and sensibility test estimates and the SROC curve.

Study design (S): In vivo studies—observational studies or clinical trials—comparing the diagnostic accuracy of both pulp vitality and pulp sensibility tests in assessing pulpal health in permanent teeth.

### 2.4. Exclusion Criteria

- Articles published in non-English language.
- Nonclinical studies, in vitro studies and animal studies.
- Studies reporting about a single intervention without a comparison group.
- Studies on deciduous teeth.
- Studies not fully available in the database.
- Article reporting only abstracts, of which full text articles were not available.
- Studies not reporting primary outcomes of accuracy, sensitivity and specificity as well as where primary outcomes are not possible to calculate from the given raw data.
- Case reports, case series, reviews and in-studies.

### 2.5. Screening Process

The search and screening, according to the previously established protocol, were conducted by two review authors (S.P. and A.M.P.). After the initial retrieval, duplicates were removed using Covidence software (Veritas Health Innovation, Melbourne, Australia. Available at www.covidence.org), and the titles and abstracts of all the results were screened by 2 authors (S.P. and A.M.). Full texts were retrieved for those articles that met the eligibility criteria by the same 2 authors (S.P. and A.M.). The list of excluded articles at the initial retrieval was cross-checked by all the authors and disagreements were resolved by discussing amongst all. In the second phase, the full manuscripts were read and those articles that did not meet the inclusion criteria were excluded with consensus. The level of agreement between the two reviewers, calculated by Cohen’s kappa (k), was 0.90 for titles and abstracts and 0.92 for full texts. The differences among authors/reviewers were resolved by a third author (S.M.) after discussion. Some studies included both “permanent teeth” and “deciduous teeth”. If the results for the subset of permanent teeth of such studies were exclusively presented and met the eligibility criteria, they were considered for quantitative synthesis. A study was excluded if it was not possible to obtain separate results of individual study groups. For the clarification of doubts and missing data of the included studies, the respective authors were contacted by email.

### 2.6. Data Extraction

The following data were extracted from the included studies by two independent reviewing authors (S.M. and S.P.) using pilot-tested customized data extraction forms: study identification number, place of study, ethical approval, informed consent, funding and registration, number of operators, sample size, age of the patient, pathology of teeth, type of teeth, pulp vitality tests and pulp sensibility tests used, diagnostic accuracy outcomes assessed, authors’ conclusions. The numerical data were compiled from each study and the missing data related to true positive (TP), true negative (TN), false negative, false positive, sensitivity, specificity, positive predictive value (PPV), negative predictive value (NPV), positive and negative likelihood ratio were converted and calculated using Review Manager (RevMan web V 5.3, The Cochrane Collaboration, available at revman.cochrane.org), where appropriate.

### 2.7. Assessments of the Risk of Bias and Quality

The selected studies were submitted to the QUADAS-2 (Bristol Medical School, Bristol, UK), methodological quality assessment tool following the recommendations of Cochrane, the UK National Institute for Health and Clinical Excellence and the Agency for Healthcare Quality and Research for use in systematic reviews of diagnostic accuracy studies. Two aspects, risk of bias and applicability of concerns, were assessed by the QUADAS-2 tool based on three domains of patient selection, index test and reference standard. The fourth domain of flow and timing was also used for the assessment of the risk of bias in addition to these three domains [21].

### 2.8. Quantitative Analysis and Synthesis of the Meta-Analysis

A meta-analysis was performed according to the methods of the Cochrane DTA Handbook [22] using the MetaDTA: Diagnostic Test Accuracy Meta-Analysis v2.0 [23] and Review Manager. The vitality and sensibility tests (index test) were compared with the reference test to determine true-positive, false-positive, false-negative and true-negative values. Sensitivity, specificity, positive predictive value and negative predictive value were calculated, and a 95% confidence interval was applied where appropriate. A bivariate model parameter for the sensitivity and specificity of each test was used to calculate summary points, the confidence region and the prediction region. The bivariate type of model maintains the 2-dimensional nature of the data considering the correlation between sensitivity and specificity instead of converting sensitivity and specificity pairs from individual studies into a solo marker of diagnostic accuracy. The parameter estimates of logit sensitivity and specificity with SEs, random-effect variances in logit sensitivity and specificity and the covariance between them were used. Summary estimates of sensitivity and specificity were computed by an inverse transformation of logit estimates to the original receiver operating characteristic (ROC) scale. A bivariate summary ROC curve for vitality and sensibility tests with summary operating points and 95% confidence regions was plotted using logit sensitivity and specificity estimates and their respective variances [24].

## 3. Results

### 3.1. Literature Search

**Figure 1:**
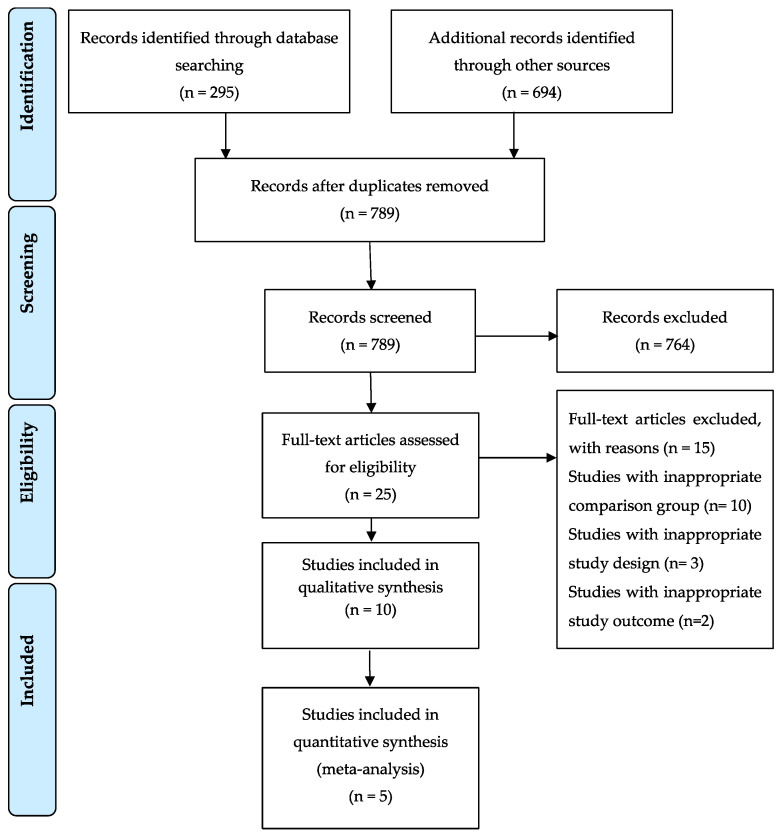
PRISMA flow diagram.

The initial electronic database search resulted in a total of 989 titles (PubMed/MEDLINE and Cochrane library resulted in 295 titles and Google Scholar resulted in 694 titles, hand searching of the reference lists of the selected studies did not deliver additional papers) and, after removal of duplicates, 789 titles remained. Out of these 789 articles, 764 were removed at the initial screening after reading the titles and abstracts. Following examination and discussion by the reviewers, 25 articles were selected for full-text evaluation. Following pre-screening and application of the eligibility criteria, 10 studies with an inappropriate comparison group, 3 with an inappropriate study design and 2 with an inappropriate study outcome were included in the qualitative analysis, while 5 studies were included in the meta-analysis. Figure 1 depicts a flowchart of the search results.

The general characteristics of 10 studies [7,10,11,12,16,25,26,27,28,29] are presented in Table 2. All included studies were unicentric trials published between 2007 and 2020. Notably, five investigations were executed in India [7,12,25,27,29], one in the United Kingdom [28], one in Ohio [26], one in Iran [11], one in Australia [16] and one in Turkey [10]. All the included studies were diagnostic accuracy studies conducted on permanent teeth. The age of the participants ranged from 6–74 years. In nine studies [10,11,12,16,25,27,28,29], ethical approval was obtained, whereas informed consent was gained in eight studies [7,10,11,12,25,26,27,28]. Only three studies [7,10,16] provided financing information, and only one study [28] was a registered clinical trial. Pulp vitality was examined utilizing PO in six investigations [7,11,12,25,27,29] and LDF in three investigations [16,26,28], whereas in one study [10], both PO and LDF were employed to assess pulp vitality. In all the studies which assessed pulp vitality using PO, the systemic oxygen saturation (SaO_2_) of the left index finger was measured first, which served as the control for the SaO_2_ values measured on the teeth. The results of the vitality tests were compared to the sensibility test, including the pain response to cold [7,11,12,16,25,26,27,28,29], electrical pulp tests [7,10,11,12,16,25,26,27,28,29] and heat test [11,12,29]. The brands and models of the PO, LDF and EPT differed among investigations, as did the method utilized in CT and HT. The cold test was assessed using Endo-Ice refrigerant spray/1, 1, 1, 2-tetrafluoroethane spray [7,11,12,16,25,27,29] and ethyl chloride [28] while for heat tests, a rubber cup [12] and gutta-percha [12,23] were used. The selected studies either reported values for sensitivity and specificity or provided sufficient data to enable calculations of TP, TN, FP, FN, sensitivity and specificity and are presented in Table 3. The PPV, NPV, positive and negative likelihood ratio (LR+, LR-), prevalence and diagnostic odds ratio for included studies along with the pooled estimates for PO, EPT, CT and HT are presented in Table 2.

### 3.2. Quality Assessment of the Included Studies

**Figure 2:**
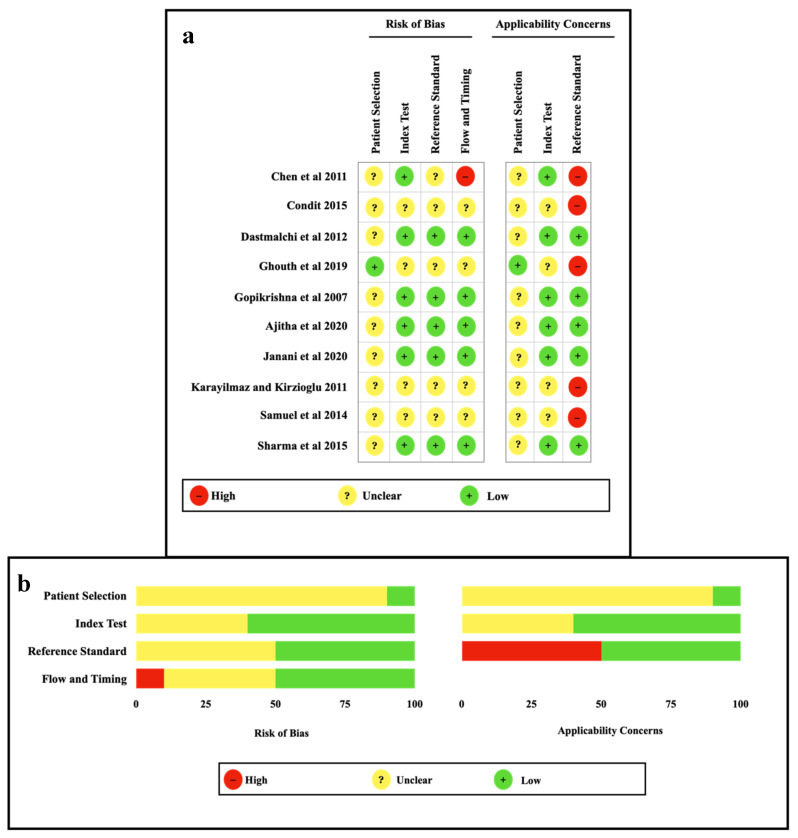
The quality assessment results of the included studies [7,10,11,12,16,25,26,27,28,29]. (**a**) Individual studies and (**b**) Within studies.

The quality assessment results of the included studies are presented in Figure 2. With the exception of one study, all other studies were found to have an unclear risk of bias; since convenience sampling was applied in all, the description of patients before inclusion in the studies was different. The index test in the QUADAS-2 tool for six studies was associated with a low risk of bias, while the remaining four studies showed an unclear risk of bias as interpretation of results with knowledge of the results of the reference standard was not mentioned. Regarding the reference standard, five studies showed low risk of bias, and five studies were identified with unclear risk of bias as there was no mention about the reference standard test used. The flow and timing characteristics were associated with a low risk of bias for five studies, and four studies and one study were identified as unclear and high risk of bias, respectively.

### 3.3. Quantitative Analysis and Synthesis of Results

A quantitative synthesis (meta-analysis) was carried out on the selected five studies [7,10,11,27,29]. In the study by Janani et al. [12] for PO and the studies which assessed the diagnostic accuracy for LDF [10,16,26,28], TP, FP, TN, TP values cannot be calculated from the given data, hence the studies were not included in the meta-analysis and only qualitative analysis was carried out (Table 3). The PO was compared with EPT, HT and CT separately. Subsequently, a total of three forest plots and summary ROC curves were made separately to calculate the sensitivity and specificity of the vitality and sensibility tests. The bivariate output box parameter estimates required for input in RevMan to produce the summary point, 95% confidence region and 95% prediction were calculated using MetaDTA software (v2.0; Shinyapps, RStudio PBC, Boston, MA, USA).

### 3.4. Diagnostic Accuracy of PO and EPT

**Figure 3:**
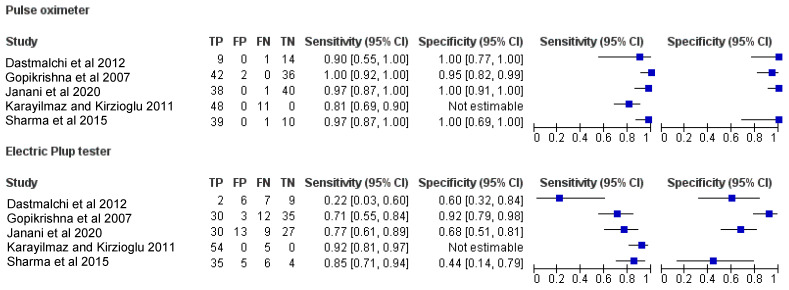
Forest plots demonstrating the sensitivity (**left**) and specificity (**right**) [7,10,11,12,27].

The pooled diagnostic accuracy values from five studies [7,10,11,27,29] for PO and EPT were obtained from the raw TP, TN, FP and FN values for each study. A summary of the pooled diagnostic accuracy values is presented in Table 2. Forest plots demonstrating the sensitivity (left) and specificity (right) of PO and EPT are presented in Figure 3.

**Figure 4:**
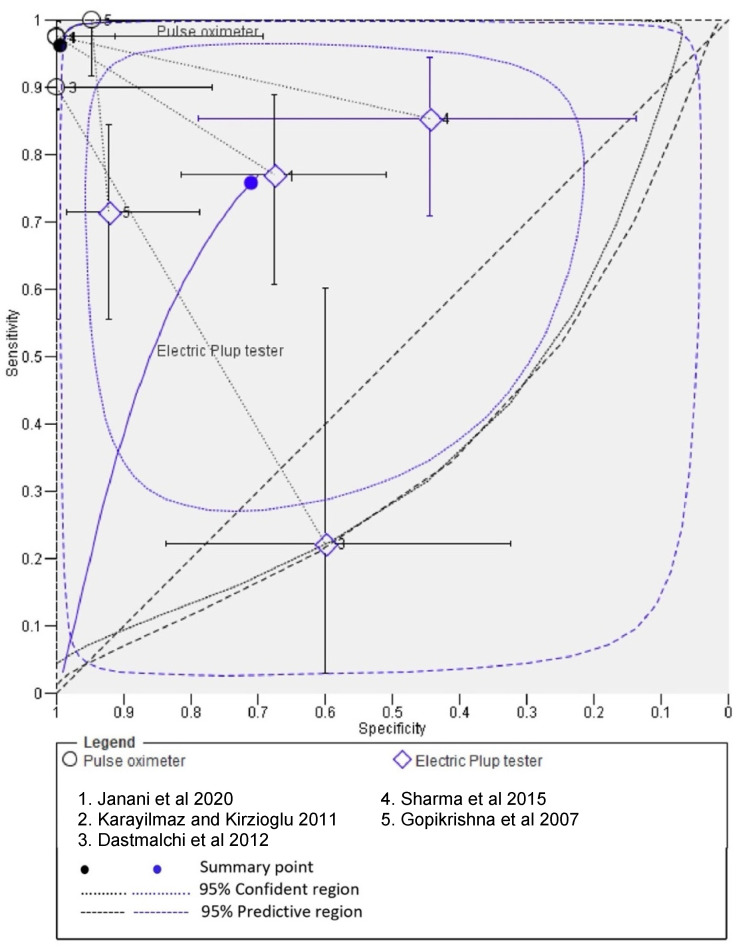
Bivariate meta-analysis of pooled mean sensitivity with PO and EPT [6,10,11,12,27].

Bivariate meta-analysis demonstrated a higher pooled mean sensitivity with PO (93%; 95% confidence interval, 88.0% to 96.0%) compared with EPT (79.0%; 95% confidence interval, 73.0% to 85.0%). A higher pooled mean specificity with PO (98%; 95% confidence interval, 93% to 100%) was also observed as compared to EPT (74.0%; 95% confidence interval, 64.0% to 82.0%). Figure 4 illustrates the calculated summary ROC curves, including the summary operating points for sensitivity and specificity and 95% confidence ellipsoids.

### 3.5. Diagnostic Accuracy of PO and CT

**Figure 5:**
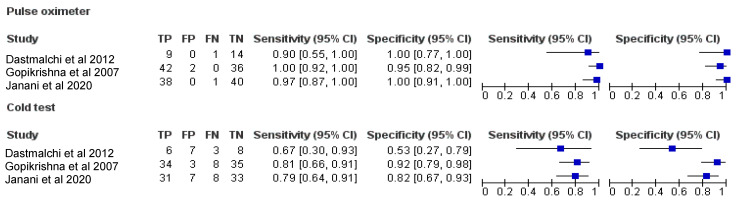
Forest plots demonstrating the sensitivity (**left**) and specificity (**right**) of PO and CT [7,11,29].

The pooled diagnostic accuracy values from three studies [7,11,29] for PO and CT were obtained from the raw TP, TN, FP and FN values for each study. A summary of the pooled diagnostic accuracy values is presented in Table 2. Forest plots demonstrating the sensitivity (left) and specificity (right) of PO and CT are presented in Figure 5.

**Figure 6:**
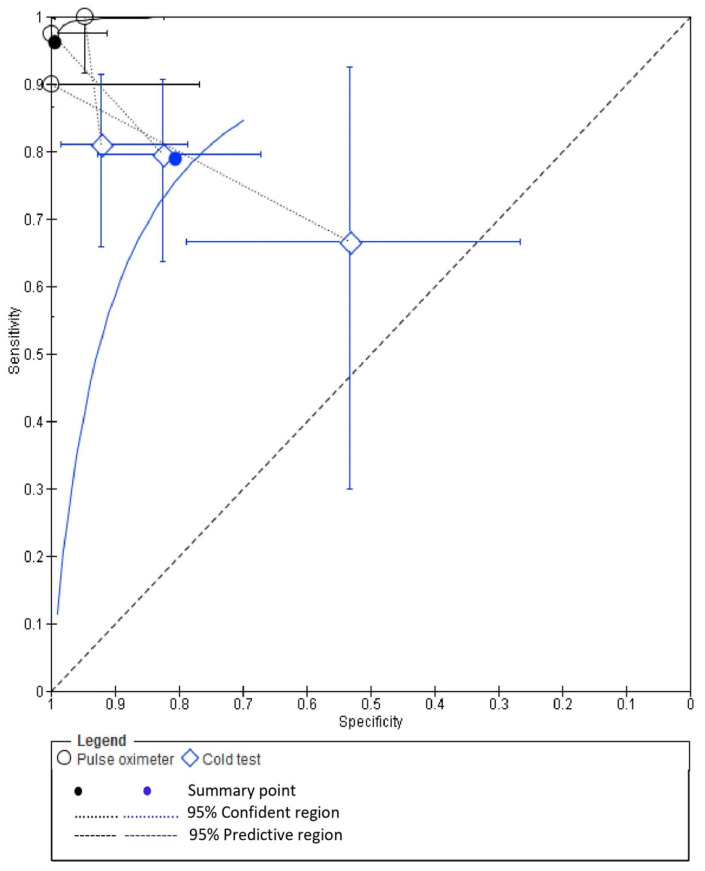
Bivariate meta-analysis of pooled mean sensitivity with PO and CT.

Bivariate meta-analysis (Figure 6) demonstrated a higher pooled mean sensitivity with PO (98%; 95% confidence interval, 92.0% to 100.0%) compared with CT (79.0%; 95% confidence interval, 69.0% to 87.0%). A higher pooled mean specificity with PO (98%; 95% confidence interval, 92.0% to 100.0%) was also observed as compared to CT (82.0%; 95% confidence interval, 72.0% to 89.0%).

### 3.6. Diagnostic Accuracy of PO and EPT

**Figure 7:**
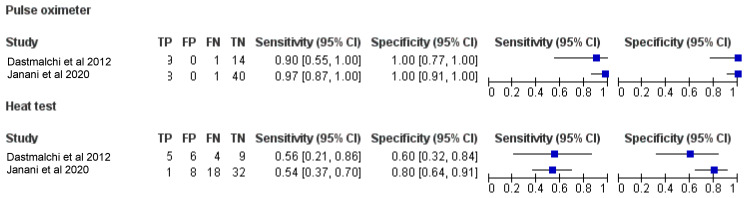
Forest plots demonstrating the sensitivity and specificity of PO and EPT [11,12].

The pooled diagnostic accuracy values from two studies [11,12] for PO and HT were obtained from the raw TP, TN, FP and FN values for each study. A summary of the pooled diagnostic accuracy values is presented in Table 2. Forest plots demonstrating the sensitivity and specificity of PO and EPT are presented in Figure 7.

**Figure 8:**
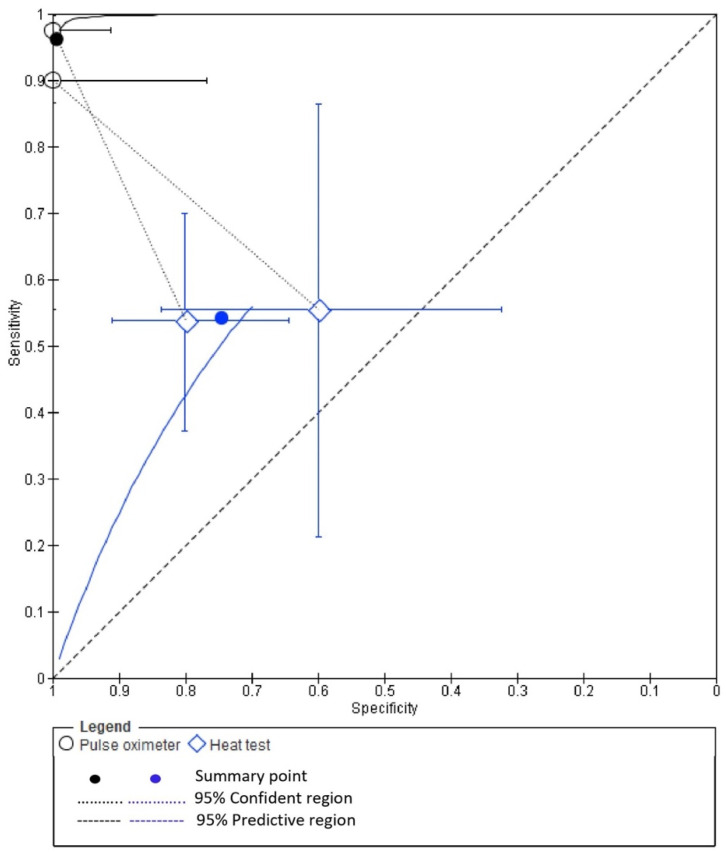
Bivariate meta-analysis pooled mean sensitivity with PO and HT.

Bivariate meta-analysis (Figure 8) demonstrated a higher pooled mean sensitivity with PO (98%; 95% confidence interval, 89.0% to 100.0%) compared with HT (54.0%; 95% confidence interval, 39.0% to 69.0%). A higher pooled mean specificity with PO (100%; 95% confidence interval, 93% to 100%) was also observed as compared to HT (75.0%; 95% confidence interval, 61.0% to 85.0%).

The summary curve and 95% prediction region cannot be computed for CT and HT as the covariance estimates were zero.

## 4. Discussion

The evaluation of the dental pulp status is essential for determining an appropriate endodontic therapy. The aim of this systematic review and meta-analysis was to evaluate the diagnostic accuracy of pulp vitality and pulp sensibility tests in assessing pulpal health of permanent teeth.

Diagnostic accuracy relates to the ability of a test to correctly identify or exclude a target condition [30]. The review included 10 clinical studies published from 2007 to 2020 conducted in various countries which directly compared both the techniques. The age of the included patients was 7–74 years of both genders. Hence, the results of this systematic review can be applicable to a varied population range and in conditions as close as possible to those observed in daily clinical practice. The clinical conditions and the methodologies applied in the studies differed considerably. Among the included studies, the patients required endodontic therapy for prosthodontic considerations or irreversible pulpitis; traumatized teeth; teeth free of any dental pathology or teeth with complete endodontic fillings, thus eliminating the risk of so-called spectrum bias implying that the study population may represent patients who would be exposed to the test in daily clinical practice [31].

In the present review, pulp vitality was assessed using PO and LDF. The CT, HT and EPT were used as pulp sensibility tests. The pulp sensibility tests evaluate the pulp’s nerve response rather than its vascularity [11]. Due to its significant resistance to inflammation, nerve tissue may remain responsive even after surrounding tissues have deteriorated, resulting in a false-positive response [12]. The presence of blood flow within the pulp is a reliable and true indicator of the pulp vitality as it reflects the degree of pulpal disease [11,12].

The overall results of the included studies demonstrated that the PO and LDF pulp vitality tests are more reliable methods in determining the actual status of the pulp in endodontics as compared to the pulp sensibility tests as all the individual studies demonstrated the same results [7,10,11,12,16,25,27,29] except in the study by Ghouth et al. [28] and Condit [26]. These studies stated that LDF was unable to differentiate between teeth with vital and nonvital pulps, showing a high probability for false results [26,28]. The studies examining the feasibility of LDF in clinical practice observed variable and uncertain results when the test conditions were not highly standardized [26,28]. Additionally, Karayilmaz and Kirzioǧlu [10] stated that the ability of PO in determining the vitality of healthy teeth was better than that of EPT, but it was inaccurate in determining the vitality of teeth with complete root canal fillings.

The sequence of pulp sensibility tests varied among individual studies. The application of EPT followed by thermal testing is a common sequence of pulp testing [32]. However, according to Pantera et al. [33], the sequence of pulp tests had no effect on the results of the tests when EPT and ethyl chloride were reversely used. Among the majority of included studies accessing accuracy of PO, custom-made specific dental probes were used which allows the maintenance of a constant path length for the light emitted from the LED and received by the photoreceptor sensor, thus enabling accurate readings [7,11,12,29]. To obtain the oxygen saturation of the tooth, Sharma et al. [27] employed an ear probe, whereas Samuel et al. [25] used a customized ear probe based on the anatomical shape of permanent incisors.

Test accuracy is estimated by comparing results of an index test with a reference standard, sometimes known as a “gold” standard, to give the number of true positives, false positives, false negatives and true negatives. The reference standard is used to verify the presence or absence of the target condition and may be a single test or a combination of tests [30,34]. Direct visual inspection during access cavity preparation was considered as a reference test in most of the studies for nonvital teeth. In the study by Ghouth et al. [28], a standardized reference standard of either pulpal extirpation or a completed root canal treatment was used. In studies assessing the accuracy of LDF, the tested tooth was paired with contralateral heathy teeth for flux comparison.

Ideally, test comparisons should focus on studies that have direct comparison with the index tests. Such direct comparisons ensure an unbiased comparison, but due to the limited availability of comparative studies, such analyses are not always feasible [34], whereas an indirect comparison uses all eligible studies that have assessed at least one of the tests of interest. However, the difference in accuracy is prone to confounding due to differences in patient and study characteristics [34]. In the quantitative synthesis of this review, direct pairwise comparison of pulp vitality and pulp sensibility tests was carried out.

The main outcome measures of this systematic review were to assess the pool estimates of sensitivity, specificity, PPV, NPV, likelihood ratio and diagnostic odds ratio of individual test groups as well as to compare the vitality and sensibility test estimates and the SROC curve.

Sensitivity represents the ability of a test to detect disease in patients who have the disease [19]. Thus, the test’s ability to identify nonvital teeth is indicated by sensitivity of a pulp vitality test. It is defined as a ratio, the number of persons with a positive test result who have the disease divided by the number of tested persons with the disease [7,35]. The total pooled sensitivity estimate of PO was 93% while the total pooled sensitivity estimates of PO paired with EPT, CT and HT were 93%, 98% and 98% respectively. The total pooled sensitivity estimates of EPT, CT and HT were 79%, 79% and 54%. Specificity, conversely, denotes the ability of a test to detect the absence of disease. It is defined as a ratio, the number of patients with negative test results without the disease divided by the number of tested patients without the disease [7,35]. The total pooled specificity estimates of PO as well as paired estimates with EPT and CT were 98% and for HT they were 100% while the total pooled specificity estimates of EPT, CT and HT were 74%, 82% and 75%, respectively.

A statistically significant difference was observed between the pooled estimates of PO as compared to EPT, CT and HT, suggesting the usefulness of PO for identifying vital teeth as well as not recommending CT and HT as a primary pulp testing method, but a combination of EPT with another thermal test can be considered. These results are similar to the study conducted by Mainkar and Kim [19] who demonstrated that PO was the most accurate pulp testing method and HPT was the least accurate while EPT has low sensitivity and high specificity, suggesting that it is less likely to correctly identify nonvital teeth, but more likely to correctly identify vital teeth.

According to the Deeks and Altman criteria, if the diagnostic odds ratio is greater than 20, with the LR+ in excess of unity and the LR- being less than unity, the results suggest that PO as compared to EPT, CT and HT is the most accurate diagnostic method in this systematic review; it shows consistently high diagnostic accuracy values from all included studies with little heterogeneity and, if possible, should be used by clinicians [36].

A bivariate random-effects model used in our meta-analysis assumes two levels of distribution of variance. First, a binomial distribution and logistics transformation of proportions preserve the shared characteristics within each study that link sensitivity and specificity, capturing the correlation between the two, as well as the absolute values observed in each study. The second level reflects the heterogeneity between studies in addition to that explained by the variability of sampling at the first level, assuming this heterogeneity is due to random study effects [24,37,38].

When the ROC curve originates from the left-hand border and reaches the top border of the ROC space, away from the 45-degree diagonal line, the test is considered to be accurate. This demonstrated that the pulse oximeter test was reliable in determining the actual pulp state [29]. The summary points on SROC curves also confirm the ability of PO to correctly classify screen negatives in presenting patients (i.e., health) as compared to EPT, CT and HT.

Intriguingly, the comparison of this study to previous English language systematic reviews [5,14,15,19] revealed some resemblances and some remarkable differences with respect to paired comparison between pulp vitality and pulp sensibility tests as well as the outcome measurements assessed. The main difference between the current and previous reviews is that a paired comparative assessment of pulp vitality and sensibility tests for vital and nonvital teeth was conducted along with their quantitative synthesis using a bivariate random-effects model [19]. The start and end of the search period also differed in the present study as compared to previous ones.

Nevertheless, the present review has some limitations. The clinical disparity among the selected studies could not be completely avoided. The sample size of the studies was small, thus lacking statistical power. Individual tooth type (incisor, canine, premolar and molar) and arch analysis were not attempted due to the limited number of tooth types included and the variation in the number of teeth in the maxilla and mandible.

It was also difficult to rule out clinical variability caused by age, gender model of PO, LDF, EPT, methodologies utilized for HT and CT, landmark selection and software capabilities. Additionally, there were few investigations on LDF, which limited its inclusion in quantitative synthesis. Furthermore, vitality tests have technical limitations, such as monitoring gingival blood flow that requires the use of a dental dam and the patient’s head to be stabilized in relation to the probe, both of which were lacking in the research methodology involved. There are no high-scoring studies for methodological validity, therefore future high-quality in vivo studies examining the diagnostic accuracy of pulp viability and pulp sensitivity testing with consistent outcome parameters should be performed.

Biocompatible and bioactive materials have recently been consistently recommended for the protection of the dentin–pulp complex due to their capacity to induce healing and regeneration of dental tissue. Their bioactivity is amongst the most beneficial properties for the maintenance and preservation of pulp vitality, supporting the use of these materials in vital dental procedures [39].

## 5. Conclusions

The current systematic review and meta-analysis indicated that, in diverse clinical situations, PO is the most accurate diagnostic tool when compared to EPT, CT and HT. Due to the lack of evidence, the diagnostic accuracy of LDF remains uncertain. However, the plurality of published endodontic studies use EPT, CT and HT as standard procedures for pulp viability as PO and LDF are not commonly accessible to all professionals and, if available, are rarely used due to their high cost and technical difficulties.

## Figures and Tables

**Table 1:** The search strategy and PICOS tool.

**Search strategy**	
**Focused** **Question**	Is there a difference in the diagnostic accuracy of pulp vitality and pulp sensibility tests in assessing pulpal health in permanent teeth?
**Search strategy**	
Population (#1)	(Human teeth [Text Word]) OR “tooth”[MeSH Terms] OR teeth [Text Word]) OR lower teeth [Text Word] OR upper teeth [Text Word] OR “molar”[MeSH Terms] OR molar [Text Word] OR posterior teeth [Text Word] OR anterior teeth [Text Word] OR premolar [Text Word] OR “incisor”[MeSH Terms] OR incisor [Text Word] OR canine [MeSH] OR Root canal [Text Word]) OR permanent teeth [Text Word])
Intervention (#2)	(‘Pulp vitality test’ [Text Word] OR Laser-Doppler flowmetry [MeSH Terms] OR Doppler-Laser Flowmetry [Text Word] OR Laser Doppler Velocimetry [Text Word] OR ultrasound Doppler flowmetry [Text Word] OR pulse oximetry [Text Word] OR thermometry [Text Word])
Comparisons (#3)	(Pulp vitality tests [Text Word] OR Thermal test [Text Word] OR Hot test [Text Word] OR Cold test [Text Word] OR electric pulp tester [Text Word])
Outcomes (#4)	(Diagnostic accuracy [Text Word] OR Sensitivity [Text Word] OR Accuracy [Text Word] OR Specificity [Text Word] OR Pulpal health [Text Word] OR Pulp vitality [Text Word])
Study design (#5)	(Clinical study [Text Word] OR Clinical trial [MeSH] OR randomized controlled studies [Text Word] OR randomized control trials [MeSH] OR randomized control clinical trial MeSH OR non-randomized control trials [Text Word] OR Quasi experimental studies [Text Word] OR before and after study design [Text Word] OR cohort studies [Text Word] OR in vivo study [Text Word] OR Cross-sectional study [Text Word])
Search Combination	#1 AND #2 AND #3 AND #4 AND #5
**Database search**	
Language	No restriction (Articles in English language or other language where English translation is possible.)
Electronic Databases	PubMed/MEDLINE, Cochrane Central Register of Controlled Trials, Web of Science, Open grey, Google scholar
Journals	Journal of Endodontics, International Endodontic Journal, Australian Endodontic Journal, Clinical Oral Investigations, Journal of Conservative Dentistry, Journal of American Dental Association
Period of Publication	Studies published between 1 January 2007 to 31 December 2020.

**Table 2:** Study characteristics of included studies.

Study ID	Place of Study	Sample Size Teeth/Patient	Age Range (Years)	Pathology of Teeth	Type of Teeth	Pulp Vitality Tests	Pulp Sensibility Tests	Authors’ Conclusions
Gopikrishna et al., 2007 [7]	India	80/80	Not reported	Requiring endodontic therapy for prosthodontic considerations or for irreversible pulpitis	Single-rooted incisors, canines and premolars	PO	CT EPT	Custom-built pulse oximeter dental probe is an effective, accurate and objective method of determining the vitality of permanent teeth.
Karayilmaz and Kirzioğlu, 2011 [10]	Turkey	59/51	12–18	Root canal treated	Maxillary anterior teeth	LDF PO	EPT	LDF was found to be a more reliable and effective method than PO and EPT for assessing the pulpal status of human teeth.
Dastmalchi et al., 2012 [11]	Iran	24/24	18–50	Requiring endodontic treatment for prosthodontic reasons	Single-canal mandibular premolars	PO	CT HT EPT	PO is a reliable method in determining the actual status of the pulp in endodontics; however, CT, HT and EPT are not suitable methods for pulp testing.
Janani et al., 2020 [12]	India	79	18–56	Requiring endodontic therapy	Single-rooted teeth	PO	CT HT EPT	Customized pulse oximeter sensor holder proves to be accurate, reliable and objective in assessing the actual condition of the tooth.
Chen and Abbott, 2011 [16]	Australia	121/20	18–74	Suspected or known to have pulp pathosis; previously received or currently undergoing endodontic treatment; or provisionally diagnosed as having a healthy pulp	Not reported	LDF	CT EPT	Carbon dioxide (CO_2_) crystals, EPT and LDF were reliable and the most accurate tests, but CO_2_ and EPT were less repeatable yet less time consuming than LDF.
Samuel et al., 2014 [25]	India	120/30	7–18	Free of any dental pathology	Permanent maxillary central and lateral incisors	PO	CT EPT	In young children, PO method was found to be as accurate as cold test but large variations were seen in electric pulp test.
Condit, 2015 [26]	Columbus, US	85	6–16	Traumatized teeth	Maxillary central and lateral incisors	LDF	CT EPT	LDF could not distinguish between healthy and necrotic pulp tissue among traumatized teeth.
Sharma et al., 2015 [27]	India	Not reported	4–15	Requiring endodontic therapy	Not reported	PO	EPT	PO is an objective, very sensitive and noninvasive method that can be used as a routine method for assessing the pulp vitality in primary, young permanent and mature permanent teeth.
Ghouth et al., 2019 [28]	UK	37	8–16	Root canal treated	Permanent anterior teeth	LDF	CT EPT	LDF was unable to differentiate between teeth with vital and nonvital pulps in children between the ages of 8 and 16 years, with an acceptable level of confidence.
Ajitha et al., 2020 [29]	India	30	18–50	Requiring endodontic therapy indicative of irreversible pulpitis	Single-canal incisors, canine and mandibular premolar teeth	PO	CT HT EPT	The use of custom-made holder is effective in placement of sensor probe onto the tooth surface. It aided in evaluating the actual pulp status by producing accurate interpretation of results.

**Table 3:** Diagnostic accuracy of pulp vitality and sensibility tests for the studies included in meta-analysis.

Index Test	Study Id	TP	FP	FN	TN	Sensitivity	Specificity	PPV	NPV	LR+	LR−	Prevalence	Diagnostic Odds Ratio
PO	Gopikrishna et al., 2007 [7]	42	2	0	36	1.00 [0.92, 1.00]	0.95 [0.82, 0.99]	0.9545	1.0000	19.0000	0.0000	0.5250	0
	Karayilmaz and Kirzioğlu, 2011 [10]	48	0	11	0	0.81 [0.69, 0.90]	Not estimable	1.0000	0.0000	-	-	1.0000	-
	Dastmalchi et al., 2012 [11]	9	0	1	14	0.90 [0.55, 1.00]	1.00 [0.77, 1.00]	1.0000	0.9333	-	0.1000	0.4167	-
	Sharma et al., 2015 [27]	39	0	1	10	0.97 [0.87, 1.00]	1.00 [0.69, 1.00]	1.0000	0.9091	-	0.0250	0.8000	-
	Ajitha et al., 2020 [29]	38	0	1	40	0.97 [0.87, 1.00]	1.00 [0.91, 1.00]	1.0000	0.9756	-	0.0256	0.4937	-
	Total pooled estimates	176	2	14	100	0.93 [0.88, 0.96]	0.98 [0.93, 1.00]	0.98	0.87	47.24	0.075	0.65	628.5
	For comparison with EPT *	176	2	14	100	0.93 [0.88, 0.96]	0.98 [0.93, 1.00]	0.98	0.87	47.24	0.075	0.65	628.5
	For comparison with CT *	89	2	2	90	0.98 [0.92, 1.00]	0.98 [0.92, 1.00]	0.97	0.97	44.98	0.02	0.49	2249
	For comparison with HT *	47	0	1	54	0.98 [0.89, 1.00]	1.00 [0.93, 1.00]	1.00	0.98	-	0.02	0.47	-
EPT	Gopikrishna et al., 2007 [7]	30	3	12	35	0.71 [0.55, 0.84]	0.92 [0.79, 0.98]	0.9091	0.7447	9.0476	0.3102	0.5250	29.16
	Karayilmaz and Kirzioğlu, 2011 [10]	54	0	5	0	0.92 [0.81, 0.97]	Not estimable	1.0000	0.0000	-	-	1.0000	-
	Dastmalchi et al., 2012 [11]	2	6	7	9	0.22 [0.03, 0.60]	0.60 [0.32, 0.84]	0.2500	0.5625	0.5556	1.2963	0.3750	0.43
	Sharma et al., 2015 [27]	35	5	6	4	0.85 [0.71, 0.94]	0.44 [0.14, 0.79]	0.8750	0.4000	1.5366	0.3293	0.8200	4.66
	Ajitha et al., 2020 [29]	30	13	9	27	0.77 [0.61, 0.89]	0.68 [0.51, 0.81]	0.6977	0.7500	2.3669	0.3419	0.4937	6.92
	Total pooled estimates	151	27	39	75	0.79 [0.73, 0.85]	0.74 [0.64, 0.82]	0.8483	0.6579	3.0023	0.2792	0.650	10.75
CT	Gopikrishna et al., 2007 [7]	34	3	8	35	0.81 [0.66, 0.91]	0.92 [0.79, 0.98]	0.9189	0.8140	10.2540	0.2068	0.5250	49.58
	Dastmalchi et al., 2012 [11]	6	7	3	8	0.67 [0.30, 0.93]	0.53 [0.27, 0.79]	0.4615	0.7273	1.4286	0.6250	0.3750	2.28
	Ajitha et al., 2020 [29]	31	7	8	33	0.79 [0.64, 0.91]	0.82 [0.67, 0.93]	0.8158	0.8049	4.5421	0.2486	0.4937	18.27
	Total pooled estimates	71	17	19	76	0.79 [0.69, 0.87]	0.82 [0.72, 0.89]	0.81	0.80	4.31	0.25	0.49	17.24
HT	Dastmalchi et al., 2012 [11]	5	6	4	9	0.56 [0.21, 0.86]	0.60 [0.32, 0.84]	0.4545	0.6923	1.3889	0.7407	0.3750	1.87
	Ajitha et al., 2020 [29]	21	8	18	32	0.54 [0.37, 0.70]	0.80 [0.64, 0.91]	0.7241	0.6400	2.6923	0.5769	0.4937	4.66
	Total pooled estimates	26	14	22	41	0.54 [0.39, 0.69]	0.75 [0.61, 0.85]	0.65	0.65	2.12	0.61	0.46	3.47

* Only studies with comparisons included.

## Data Availability

The reported systematic review and meta-analysis is registered with PROSPERO reg no.: CRD42020213741 available at https://www.crd.york.ac.uk/prospero/display_record.php?ID=CRD42020213741.
